# ToxPi Graphical User Interface 2.0: Dynamic exploration, visualization, and sharing of integrated data models

**DOI:** 10.1186/s12859-018-2089-2

**Published:** 2018-03-05

**Authors:** Skylar W. Marvel, Kimberly To, Fabian A. Grimm, Fred A. Wright, Ivan Rusyn, David M. Reif

**Affiliations:** 1 Bioinformatics Research Center, Center for Human Health and the Environment, Department of Biological Sciences, North Carolina State University, Box 7566, 1 Lampe Drive, Raleigh, NC 27695 USA; 2 Department of Veterinary Integrative Biosciences, Texas A&M University, College Station, TX USA

**Keywords:** Visual analytics, Software; systems biology, Risk assessment, Data integration, Graphical user interface

## Abstract

**Background:**

Drawing integrated conclusions from diverse source data requires synthesis across multiple types of information. The ToxPi (Toxicological Prioritization Index) is an analytical framework that was developed to enable integration of multiple sources of evidence by transforming data into integrated, visual profiles. Methodological improvements have advanced ToxPi and expanded its applicability, necessitating a new, consolidated software platform to provide functionality, while preserving flexibility for future updates.

**Results:**

We detail the implementation of a new graphical user interface for ToxPi (Toxicological Prioritization Index) that provides interactive visualization, analysis, reporting, and portability. The interface is deployed as a stand-alone, platform-independent Java application, with a modular design to accommodate inclusion of future analytics. The new ToxPi interface introduces several features, from flexible data import formats (including legacy formats that permit backward compatibility) to similarity-based clustering to options for high-resolution graphical output.

**Conclusions:**

We present the new ToxPi interface for dynamic exploration, visualization, and sharing of integrated data models. The ToxPi interface is freely-available as a single compressed download that includes the main Java executable, all libraries, example data files, and a complete user manual from http://toxpi.org.

## Background

With modern data generation technologies producing massive volumes of information, effectively communicating results presents a daunting challenge. This is especially true when data must be integrated from diverse sources to present results within a holistic, systems-level context. Moreover, the intended audience for such integrative experiments may represent several scientific disciplines, policy makers, and even the general public. For these transdisciplinary situations, interactive visualization offers the most effective means of communication [1].

Here, we present an interface for the ToxPi (Toxicological Prioritization Index) framework that provides interactive visualization, analysis, reporting, and portability. ToxPi is a flexible, decision-support tool that was developed to enable integration of multiple sources of evidence (e.g. information on the hazard, safety, and exposure of environmental chemicals) by transforming data into transparent, visual rankings [2]. The ToxPi profiles effectively communicate results from the increasingly high-dimensional data used in modern systems biology, biomedical research, and the environmental health sciences. This approach can be tailored to diverse prioritization tasks, risk/decision-assessment needs, and interactive visualizations [3–7].

**Fig. 1:**
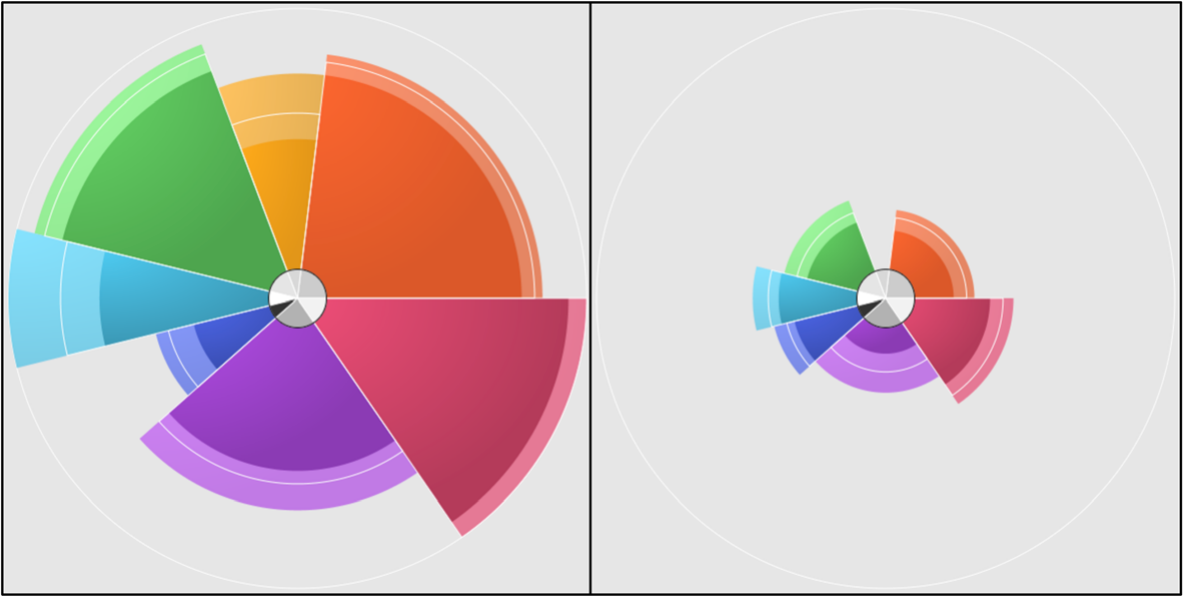
Anatomy of a ToxPi profile. Profiles for two example chemicals (simulated data) are shown for a model combining source data into 7 slices. For each slice, the distance that the arc extends from the origin is proportional to its relative evidence of concern (e.g. longer = greater risk), and the radial angle (width) indicates its weight in the overall model. The optional confidence intervals (upper and lower 95%) are indicated as the lighter-shaded area at the boundary of each slice arc. The inner circle indicates the percentage of missing data (darker = higher missingness) in each slice. In this example, the profile on the left has a higher overall priority score (and would be ranked higher in the distributional rank plot in Fig. 2) than the profile on the right

In the chemical safety realm, ToxPi has been popular for communicating risk prioritization and profiling information between scientists, regulators, stakeholders, and the general public. The ToxPi yields an explicit, prioritized order of chemicals, as well as a visualization of the underlying, weight-of-evidence scheme that specifies the contribution of each data source to a chemical’s activity or risk profile (Fig. 1). As a result of this transparency and accessibility, ToxPi has been featured in reports and monographs by the U.S. National Academy of Sciences [8–10], the World Health Organization’s International Agency for Research on Cancer [11–13], and as part of the toolkit used by the U.S. Environmental Protection Agency for a number of data visualization dashboards [14].

As methodological advancements have continued to improve ToxPi and expand its application domain, there exists a need for a consolidated software platform to provide functionality while allowing flexibility for future improvement. While the first-generation graphical user interface (GUI) for ToxPi was robust software that has been heavily downloaded and used across several disciplines [15], its flexibility was limited by a cumbersome data input format and an architecture built for consistency over modularity. The interface introduced here capitalizes on progress in the software development community to create a complete product that can import data in several formats (from a basic matrix to more fully-annotated data structures), then interactively build models and analyze results in a manner (such as profile-similarity grouping) that has not yet been possible. Most importantly, the new interface was purpose-built to accommodate expansion with new modules and upgrades to existing methods. Several new modules are described in the following sections, covering functions such as similarity-based clustering of ToxPi profiles. We have also included functionality to ensure backward compatibility so that legacy data models can still be used. In the following sections, we describe how this new ToxPi software transitions the interface into a fully-featured analysis suite that allows dynamic exploration of data.

## Implementation

This version of the ToxPi interface was developed using the JavaFX platform for a modern look-and-feel (versus the older Java Swing that was used for the first-generation GUI). The main ToxPi functions have been recoded to incorporate published methodological updates for scaling overall scores into a consistent [0–1] interval that facilitates comparison across models [16]. For users interested in source code for calculating ToxPi scores, a complete set of R code and data files are freely-available as Supplemental Material with Auerbach et al. [17]. The internal data structures have been reorganized to facilitate new analysis modules. While this new software permits additional flexibility in input file formats, we have provided for backward compatibility of data models created for the previous version. The clustering modules were ported to Java from R source code for the functions hclust and kmeans [18], using the slice-wise ToxPi scores as feature vectors. Hierarchical clustering results are visualized using a custom Dendrogram.java class. The ability to save figures in high-resolution SVG and PDF formats has been added.

The software is distributed as a single ZIP file for download. The compressed ZIP includes the main JAR executable, libraries needed, example data files, and a user manual. Users need only to open the main *ToxPi.jar* executable to get started. Most users will already have Java installed and configured for regular updates, though it is a free download for any operating system [19]. An illustrated, step-by-step manual is included with the ToxPi download. The manual includes a “Quick-Start” section for users who want to dive directly into the data and learn by example. For more detail, we discuss implementation of each ToxPi functional module in the following sections. Although any type of data may be used, the description of each module refers to a data set of distinct chemical entities (rows) that have measurements across a set of bioassays or other chemical measurements/metrics (columns).

### Data import module

The Data Import module (in the *File Selection* tab) can handle a range of data structures, which are input as flat CSV files. The most basic data structure that can be imported is a simple matrix that includes row and column names. More sophisticated data structures can be imported that include header information for rows (e.g. chemical identifiers or classes) and/or columns (e.g. assay descriptors or ToxPi model information). The most complete data structure matches the file format that is output by the software following model building. This complete data structure can be shared with colleagues or published alongside results to allow users to load (and manipulate) ToxPi models, including all apportionment, coloration, and weighting choices.

Multiple input files may be uploaded. The software will present all available chemicals (i.e. instances) and assays (i.e. metrics). The user can then choose to include all instances or a subset, for which common metrics are presented. Missing data for any row-column pair are indicated by ‘NA’ and will be ignored, along with any negative numerical values. Optionally, users can load a preconceived model by selecting the *Recreate From File* button.

### Model construction module

The essence of a ToxPi model is the recombination of singular data sources (“components” or “metrics”) into explicitly-weighted slices. The Model Construction module (in the *Slices* tab) provides options for building slices as recombinations of one or more assays. Functionality permits inclusion/removal of assays on a click-by-click basis or in batches via text search, selection of data source type, or *Add/Remove All*. Options for scaling each slice are presented alongside summary statistics. As slices are created and weighted, the legend image can be toggled on/off to dynamically display the current state of the overall ToxPi model. While the software does not set a maximum on the number of slices, an excessive number may reduce the visual effectiveness of resulting ToxPi profiles. Slices can be rearranged or recolored in a one-by-one or batch fashion. For users who have loaded a preconceived model, the Model Construction module will be initiated with all parameters of that model, which users can choose to modify as above.

### Visualization of the results module

**Fig. 2:**
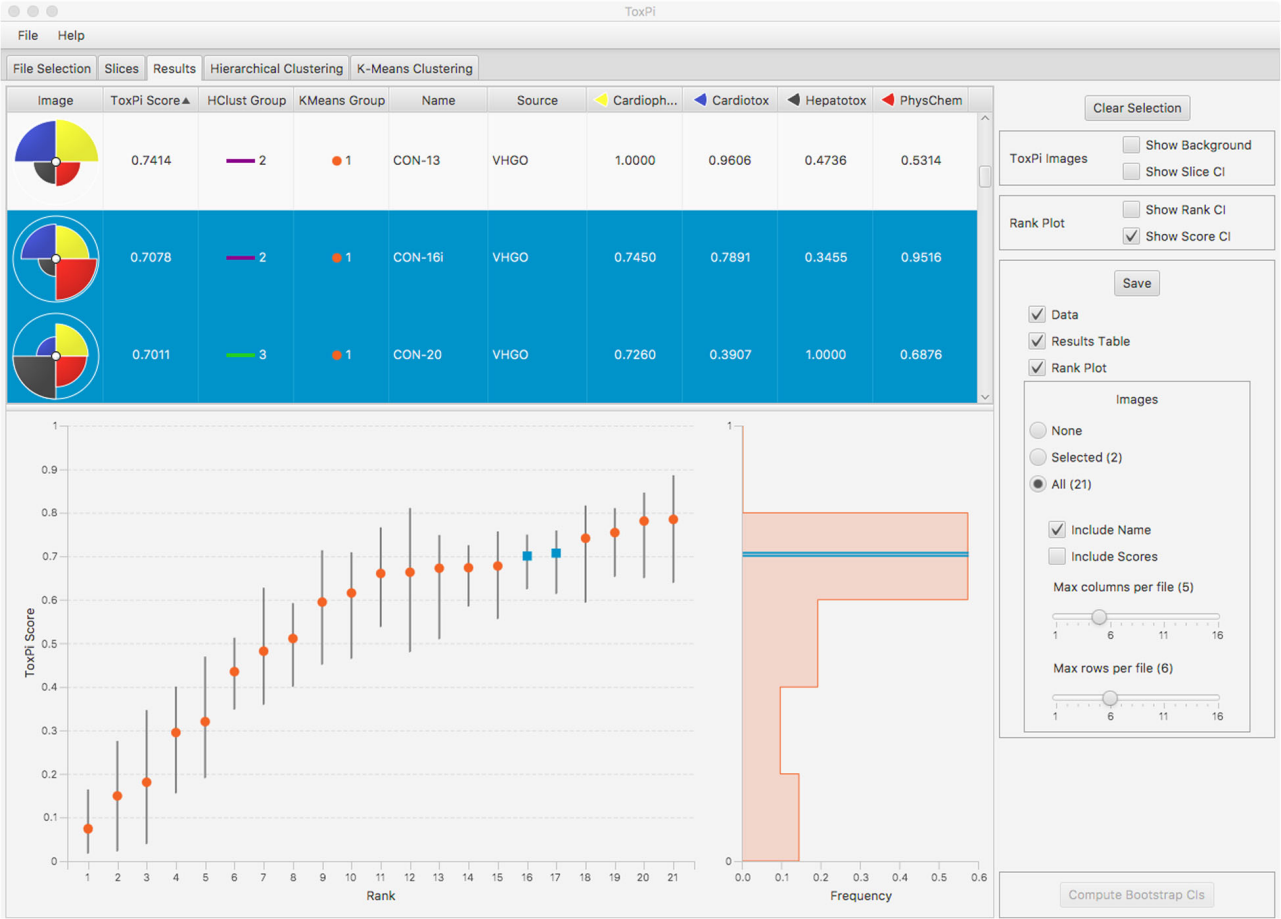
Results tab. Each row in the table (upper panel) displays information for a single entity, including the ToxPi profile image, overall score, cluster group membership, entity name and source, and scores for each slice. Each column can be sorted individually or as part of a hierarchical sort with additional columns. The two highlighted chemicals are annotated on the rank plot and histogram (lower panel) as blue dots and lines, respectively. Options for display of contrasting backgrounds or confidence intervals on profile images and rank- or score-wise confidence intervals on the rank plot will be reflected in saved output files (right panel). Additional details and capabilities are described in the User Manual that is included with the download

The Results module displays an interactive table of information on individual chemical profiles, the global distribution of scores (rank plot and associated histogram), options for estimating confidence intervals, and customization choices for output files (Fig. 2). Individual chemicals can be selected for zoomed-in viewing of profiles and score details (Fig. 1). The entire results table can be sorted by selecting any column, including chemical name, data source, overall score, slice-wise score, or cluster membership (see following sections). Hierarchical sorting is possible by selecting additional columns in a preferred order. Chemical sets can be manually selected or batch selected following resorting. Selected sets will be highlighted in the global distribution plot. Selected sets or results for all chemicals can be written to file as shareable input data (CSV), statistical results tables (CSV), rank plots (PNG), and customized arrangements of ToxPi profile arrays (PDF, PNG, SVG).

### Hierarchical clustering module

**Fig. 3:**
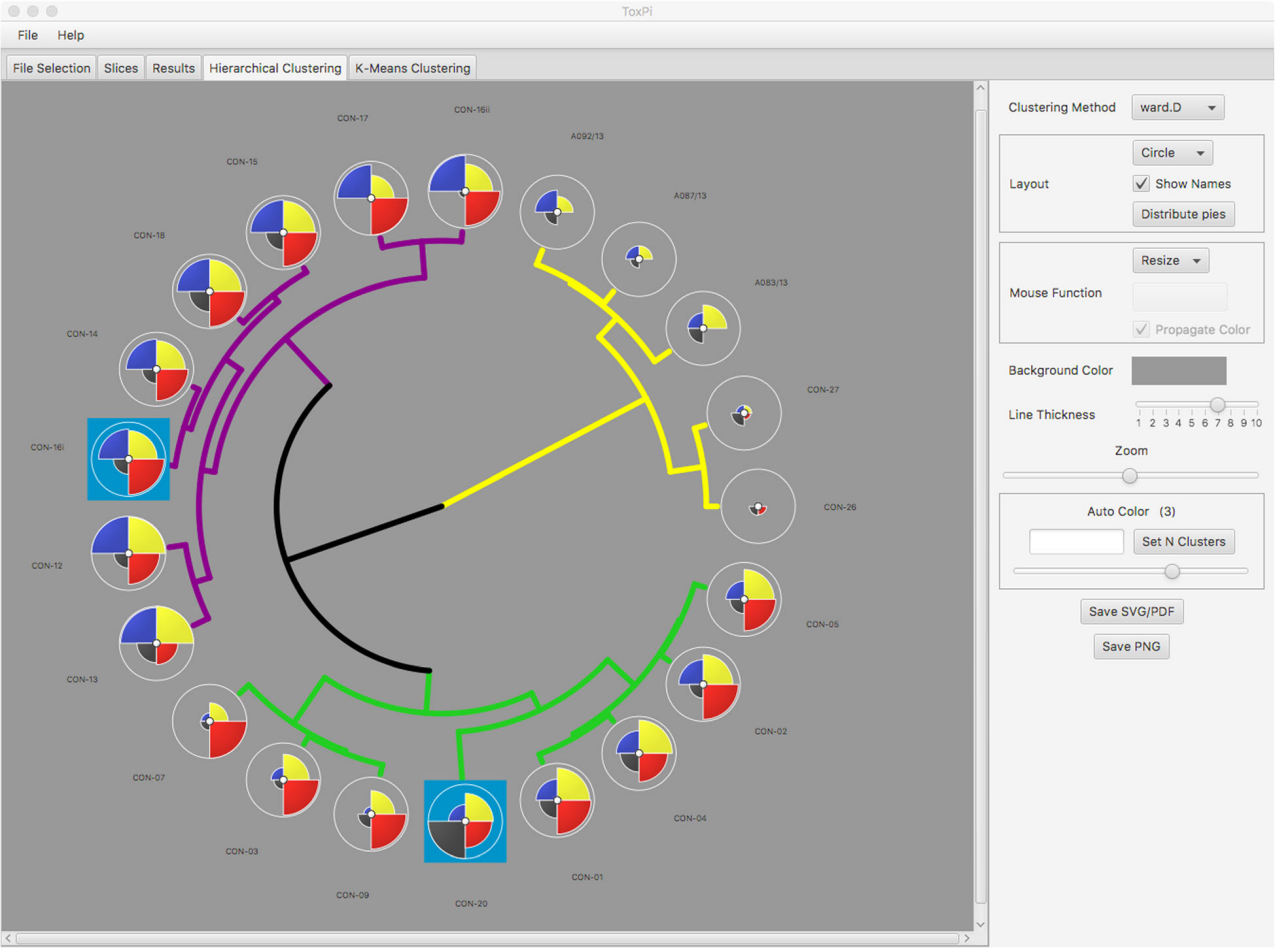
Hierarchical Clustering tab. The highlighted chemicals from *Results* are shaded in blue in the main plot area (left panel) of the *Hierarchical Clustering* tab. The entire dendrogram can be saved using the *Save SVG/PDF* or *Save PNG* buttons. Using the visualization options (right panel), a circular dendrogram was selected, the *Distribute Pies* button optimized the spacing to show detail for each ToxPi*,* and clusters were automatically colored by branch depth. Additional details and capabilities are described in the User Manual that is included with the download

The Hierarchical Clustering module provides options for organizing ToxPi profiles into clusters based upon similarity (Fig. 3), rather than the default sorting by overall priority score (rank). The cluster dendrograms are drawn using one of six hierarchical clustering methods, with ToxPi profiles for individual entities (e.g. chemicals) at each leaf. This module is intended to aid results interpretation and can be used for assessing profile similarity, as in chemical read-across applications [20]. Several options are provided to adjust clustering parameters and display properties. The choices of clustering methods correspond to those available with the R function hclust, using Euclidean distance. For users interested in alternative clustering approaches or additional parameter control, the manual provides R code for replication of GUI results. Because diverse layout options (e.g. circle versus hanging dendrogram) and dataset sizes demand different display requirements, options for manual or automatic optimization of the display region are provided. Clusters can be defined in an automated (i.e. top-down) fashion or by selecting subsets of dendrogram branches. Clusters defined will be dynamically updated in the Results module. The save-to-file buttons will write all features of the current display state to an external file (PDF, PNG, SVG).

### K-means clustering module

The *K-Means Clustering* module uses agglomerative clustering to organizing ToxPi profiles into clusters based upon similarity, rather than the default sorting by overall priority score (rank). The clusters are plotted on a principal components analysis (PCA) coordinate field, where each point represents the ToxPi for a single chemical [15]. The points are colored and shaped according the user-defined number of groups (*nClusters*). Clusters defined will be dynamically updated in the Results module. This module assesses profile similarity by an agglomerative, bottom-up approach, as opposed to the *Hierarchical Clustering* module’s top-down, divisive approach. The implementation is a Java port of the R function kmeans using the “Hartigan-Wong” algorithm. The algorithm is run *nStart* times with different starting cluster locations, with the best clustering result chosen as that having the smallest within-group sum of squares. The seed of the random number generator (*Seed*) can be specified in order to replicate results. For display and exploration, hovering the mouse over any point will bring up a tool tip with information on that chemical, and options are provided to flip the orientation of one or both principal components. The save-to-file buttons will write all features of the current display state to an external file (PNG).

### Dynamic module interaction

All modules interact dynamically so that changes flow through to any given tab. This assures that users conduct a consistent analysis while proceeding through the modules of their choice. For example, selections made in either of the two clustering modules will flow to the Results module. Cluster groups (if any are defined) are presented in the Results table. The color and shape of points in the Results rank plot will match those in the K-Means Cluster module (with default circles used if no clusters have been defined). Individual chemicals can be highlighted across modules by selecting individual rows in the Results table, points in the Results rank plot, ToxPi leaves in the Hierarchical Clustering dendrogram, or points in the K-Means Clustering plot. Chemical selections can also be made in batch for each module (Shift-click). Selections can be captured in output files by choosing the ‘Selected’ radio button. This dynamic interaction allows users to explore by combinations of prior knowledge or hypotheses (Results table), priority rank (Results rank plot), or either of the clustering modules, then share results accordingly.

## Results and discussion

For software testing and illustrating functionality, the ToxPi distribution includes several example data files. Four data files represent reconfigurations of a single data set into each of the four basic input file formats (see detailed illustrations in the user manual). The remaining three data files are examples representative of published ToxPi applications, from small (50 chemicals), to medium (300 chemicals), to large (1000 chemicals). The example data files of different sizes were used to externally test the software on machines running recent versions of either Windows or Mac OSX. Results showed that this software, designed for dynamic user interaction, completes most functions immediately. A progress bar is provided for the initial computation of ToxPi indices and (optional) estimation of bootstrap confidence intervals, which elicit a momentary pause for data files of 1000 entities or higher.

To illustrate how this software can recapitulate published models and offer new analytics, we used the data published by Grimm et al. [20]. Briefly, data were collected on a set of petroleum substances broadly categorized as substances of Unknown or Variable composition, Complex reaction substances and Biological materials (UVCBs), which present a major categorization challenge for chemical regulatory agencies. The published ToxPi model recombined concentration-response data from in vitro screening assays with physico-chemical characteristics toward the goal of generating more informed groupings and bioactivity rankings of complex petroleum substances. The data were apportioned into four equally-weighted ToxPi slices, representing eight Cardiophysiology measures (yellow slice), three Cardiotox measures (blue slice), five Hepatotox measures (dark gray slice), and two PhysChem descriptors (red slice).

Figure 2 is a screenshot of the Results module, following direct import of the model from Grimm et al. [20]. Two chemicals having similar scores were highlighted by selecting adjacent ranks in the rank plot. The selections (CON-16i and CON-20) are propagated throughout the *Results* tab as lines on the score histogram, blue shading in the table, and population of the *Selected* button in *Save* options. The selections are also propagated as highlighted ToxPi leaves in the *Hierarchical Clustering* tab and highlighted points in the *K-Means Clustering* tab. Figure 3 shows the selected chemicals as highlighted leaves of a circular cluster dendrogram. The clustering, wherein the two highlighted profiles appear in different branches, illustrates how chemicals of adjacent priority rank may have different reasons (i.e. data sources) driving their overall scores. The *Auto Color* option has been set to emphasize the three main branches of the dendrogram. The new software clusters bioactivity profiles of UVCBs in a meaningful manner, i.e. according to manufacturing streams with similar physico-chemical properties that include Straight Run Gas Oils (SRGOs: CON-01, − 02, − 03, − 04, and − 05), Other Gas Oils (OGOs: CON-07 and -09), and Vacuum & Hydrotreated Gas Oils (VHGOs: Con-12, − 13, − 14, − 15, −16i, −16ii, − 17, − 18, and − 20). These three gas oil groups are in a distinct branch from the more complex Residual Aromatic Extracts (CON-26 and -27) and Heavy Fuel Oils (A083/13, A087/13, and A092/13), thereby exemplifying that differences in chemical composition of UVCBs are reflected in profiles of their biological characteristics. It should also be noted that ToxPi analysis resulted in clustering of one of the VHGOs (CON-20) with SRGOs and OGOs, thereby indicating that this particular UVCB biologically, and possibly chemically, might be a better fit for one of these groups than its manufacturing-denoted classification. These findings are in accordance with the major groupings discussed in [20]. In the previous analysis, a separate clustering was performed on the matrix of component data values, whereas here, the clustering is explicitly linked to the ToxPi visual profiles. Together, Figs. 2 and 3 illustrate different applications, score-based ranking and similarity-based clustering, respectively, that have been linked for users by the new software.

The clustering modules are examples of how this interface will serve as a platform for expansion into additional application domains as new modules are added. One such application is the example of defining chemical groups from ToxPi clustering information [20, 21]. Chemicals having similar ToxPi profiles could be candidates for use in “read-across” to fill data gaps, where endpoint or bioactivity information for one chemical is used to predict that same information for another chemical. For large datasets, similarity neighborhoods could be defined for entire sets of chemicals. The output files of the ToxPi interface can be shared for such clustering exercises or directly compared with other cheminformatic models.

While the majority of published applications of ToxPi have focused on prioritization of chemicals, any set of entities (instances) could be compared. For example, input data could be clinical subjects, each having measures from diverse data sources including diagnostic tests, demographic information, lifestyle questionnaires, personalized exposure data, etc. This flexible software platform is agnostic as to data types, so users can exercise expert knowledge in apportioning their data into slices, rescaling if necessary, assigning weights, then evaluating results. The dynamic interaction between all modules ensures that model parameters are accurately captured, reflected in results, and saved to shareable output files to permit reproducibility.

## Conclusions

As the size and scope of modern data-generation continue to grow, methods for interpreting those data will have commensurately-increasing requirements for transparency and interactivity. This demand is driven by pressures from an interdisciplinary scientific community as well as an informed public wanting to understand decisions made for commercial and public health reasons. Transparency in data interpretation and model formulation are facilitated by interactive software tools such as ToxPi. We present the new ToxPi interface as a modular software platform that can dynamically explore data, immediately see impacts of model adjustments, share reproducible models, and communicate resulting visualizations in tandem with the underlying data.

## Availability and requirements

Project name: ToxPi.

Project home page: http://toxpi.org

Operating system(s): Platform independent.

Programming language: Java.

Other requirements: Current Java JRE (Free from http://oracle.com).

License: GNU GPL.

## Abbreviations

CSV: Comma-Separated Values (file format)
JAR: Java ARchive (file format)
PDF: Portable Document Format (file format)
PNG: Portable Network Graphics (file format)
SVG: Scalable Vector Graphics (file format)
ToxPi: Toxicological Prioritization Index
